# Hospital and Health News

**Published:** 1924-10

**Authors:** 


### HOSPITAL AND HEALTH NEWS.

### Turkish Women Doctors.

In future it will be possible for Turkish women to become doctors, the prohibition against their attending classes of the Medical Faculty at Constantinople having been removed.

### One Doctor per Thousand.

During 1923 the Medical Register recorded 2,483 new names—by far the largest entry for any one year. There is now one doctor to every 1,000 persons in the British Isles.

### Maternal Mortality.

American statistics show that there was a lower death rate of mothers from childbirth of puerperal causes in 1922 than in any year since 1916.

### Bermondsey Experiment Refused.

The Minister of Health and the L.C.C. have declined to sanction the Bermondsey Borough Council's proposal to send an experimental party of tuberculosis patients to Leysin.

### Votes for Nurses.

The Registration Officer of Southwark recently gave a decision granting the franchise to nurses engaged in hospitals and similar institutions by virtue of service within Section 3 of the Representation of the People Act.

### What is the Answer?

A member of one of the Ward Hospital Committees in the city of Leeds asks why the monthly meetings may not be held on licensed premises, which cost nothing to the fund. What is the answer?

### Hospital Used Once in Thirty Years.

The Medical Officer of Health of Sandbach states that the small-pox hospital has been used only once during the last 30 years. He thinks it a pity that it could not be utilised for sleeping sickness cases, etc.

### Income of the London.

The income of the London Hospital in the past year was £361,000, of which half resulted from the gifts received to double those conditionally promised by the donors. The expenditure was £258,000.

### That Night Nurses May Sleep.

Rubber carpet is to be laid in the corridors of the extension now being added to the Nurses' Home of the St. Marylebone Hospital. The idea is to deaden the sound and secure the night nurses from disturbance when resting during the day.

### St. Mary's Hospital Medical School.

The Annual Dinner of Past and Present Students of St. Mary's Medical School will take place at the Connaught Rooms, Great Queen Street, W.C., on October 6, at 7.0 for 7.30 p.m. Dr. E. M. Callender, C.B.E., will be in the Chair. The Hon. Secretary is Dr. Hope Gosse.

### Buchanan Hospital, St. Leonards.

To double every donation made when the Earl of Ypres opens the new ward of the Buchanan Hospital, St. Leonards, is the offer of an anonymous friend. His limit is £3,000, and if it is reached the debt upon its building will have been paid.

### New Vaccination Order.

After October 1 parents of newly-born children will not be required to apply to the vaccination offices for forms of declaration of conscientious objections to vaccination. By an order issued by the Minister of Health the forms will be handed to them by the Registrar when registering the births.

### Monkwearmouth Hospital.

The governors of Monkwearmouth Hospital have adopted a scheme for a new hospital, on the Ashville site, and Sir James Marr, Bt., is chairman of the special subscription committee. Sunderland is also in need of additional hospital beds. The Royal Infirmary has not much room for extension, and some supporters of the new scheme hope that both hospitals may co-operate in a common policy, in which case a joint appeal could be made. Hitherto attempts at amalgamation have failed, and to this failure the Monkwearmouth decision to build a new hospital has been attributed.

### All Indoor Appointments for Women.

For the first time in the history of the hospital all the indoor appointments at the Glasgow Royal Maternity and Women's Hospital have been given to women. They were secured by Dr. Ellen B. Sutherland, Dr. Mary P. MacCunn, Dr. Janet M. Stewart, and Dr. Ellen D. Morton.

### Elections to Wanstead Orphanage.

The next half-yearly election for the admission of fatherless children to the Royal Infant Orphanage, Wanstead, E. 11, will be held in November. Children under seven years of age are eligible, and applications for information and nomination papers should be addressed to the Secretary at the Orphanage at once.

### Accident Cases at Poor Law Hospitals.

The Bethnal Green Board of Guardians has adopted the chief recommendations of King Edward's Hospital Fund for the admission of accidents to Poor Law Hospitals, and trusts that the Minister of Health will not oppose the steps necessary to provide an adequately staffed casualty department at the institution.

### Boycotting Patients.

A new law has been adopted at Basle permitting any person to practise the art of healing in the canton although not a medical man. Qualified doctors therefore are reported to be boycotting the patients who have recourse to the advice and aid of the unqualified.

### Gifts to the Miller Hospital.

The Miller General Hospital for South-East London has received the gift of a house, Adar Bank, near Greenwich Park, and £12,000 for its maintenance, from the late Mr. Frederick Fountain, a Greenwich outfitter. The house, which stands in some three acres, was bequeathed for a convalescent home.

### A Continuous Decline.

The Essex County Hospital, Colchester, has suffered for four years a continuous decline in annual subscriptions. A discouraging fact is that few of the employers are maintaining the standard of 1921, when their subscriptions were based on the number of their staffs.

### Wandsworth's Good Fortune.

The Bolingbroke Hospital, Wandsworth Common, is about to add a William Shepherd Memorial Wing at a total cost of £60,000. Towards this Sir James and Lady Carmichael, of Kingston Hill Place, Kingston, have given £10,000.

### The Edith Cavell Homes of Rest.

A flower day will be held in aid of the endowment of the Edith Cavell Homes of Rest for Nurses, on Friday, October 10, in the City of Westminster. Helpers will be heartily welcomed and donations gratefully received. Communications should be sent to the Hon. Secretary, Miss W. E. Hall, at 32 North Audley Street, W. 1.

### Telephone Company's Hospital Contract.

The Army, Navy, and Air Force Hospital, Chatham, is about to be fitted with a 50-line " Relay " telephone exchange by the Relay Automatic Telephone Company of Marconi House. The contract, which was secured in competition, includes supplying a special 550 automatic telephone installation for H.M. Dockyard at Chatham.

### A New Tonic.

A new medicine of which good reports are coming in from doctors and nurses is Integar, which it is claimed touches some mainspring of endocrine balance and gives a feeling of exhilaration to those who take it regularly as a tonic. No secret is made of the ingredients, and the makers are prepared to submit their case reports for discussion at a medical meeting. Integar is sold in boxes of 24 capsules at 2s. 9d.

### New Secret Remedies Order.

An Order has been issued by the Privy Council under which secret remedies, such as chlorodyne (which contains three poisons), in addition to being labelled with the name and address of the seller, and with the word " poison," must bear on the label the name of each of the poisons and the proportion of each. The Order will be enforced a year hence.

### The Duke's Cure.

The Duchess of Devonshire, opening a bazaar at Chatsworth in connection with the Devonshire Hospital, which for many years has been the home for chronic sufferers from rheumatism, related that the Duke had recently visited Buxton for the "cure," and was so much relieved that the boot which formerly he was unable to lace was now three sizes too large for him.

### A Record Bazaar.

The total result of the Harrogate Infirmary Bazaar was £40,450. On the bazaar itself the profit was £22,750. Besides there were donations in cash and deferred donations, amounting to £15,450, to be spread over six and a half years. The expenses of the bazaar were £517, and Mr. A. W. Bain, chairman of the infirmary committee, believes the figures to be a record for a bazaar in the north of England.

### Lord Knutsford and Infirmaries.

The protests aroused by Lord Knutsford's remarks upon Poor Law Infirmaries have not ceased. He has been dosed, almost daily, with replies. We trust that the ultimate effect will be to make the Guardians more determined than ever to improve their infirmaries and abolish understaffing; not to make them disinclined to co-operate with the hospitals where they have available beds.

### Model Ward at Wembley.

The model children's ward at the Exhibition at Wembley has been placed there by the West London Hospital. It is to be found by the Arkwright Gate of the Palace of Engineering. A baby is shown under treatment, and the stand is in charge of a nurse.

### Jubilee of Royal Bath Hospital, Harrogate.

On September 3 the Royal Bath Hospital and Rawson Convalescent Home, Harrogate, celebrated its centenary. Its work is national, because it is one of the three hospitals for the treatment and investigation of rheumatism. It is interesting to note that the appeal for £12,000 made on this occasion was not for extension. The committee believes individual attention to a limited number to be preferable to an attempt to treat a host of patients.

### The Training of Public Health Workers.

Courses of training for Public Health Officers at the Royal Sanitary Institute began on September 22 with an introductory lecture by Professor Kerswood. The Sanitary Officers' courses commenced on September 24, and those for Health Visitors and Child Welfare Workers and Meat and Food Inspectors begin on September 29 and October 3 respectively. The training not only includes lectures, but practical demonstrations, visits to Welfare Centres, abattoirs, public works, etc., and the use of a library and reading-room. The courses are followed by the standard examinations of the Institute, and all further particulars can be obtained from Mr. White Wallis, at the Institute, 90 Buckingham Palace Road, S.W. 1. The National Association for the Prevention of Infant Mortality have arranged courses of elementary lectures on Infant care for crèche nurses and probationers and first graduate lectures for Health Visitors, Nurses, etc., to begin on October 2 and September 29 respectively at Carnegie House.

### What We Ought to Know About the Mind.

A series of eight lectures will be given shortly by members of the lecturing staff of the People's League of Health on "The Mind and What We Ought to Know About It." They will be delivered at the Medical Society's Headquarters, 11 Chandos Street, Cavendish Square, W. 1, beginning on

Monday, October 20, at 6 p.m., and continuing on subsequent Monday evenings till December 8. The fee for the series is 10s., and applications for tickets, which are not transferable, should be made to the Honorary Organiser, the People's League of Health, 12 Stratford Place, W. 1. The lecturers will include Sir Maurice Craig, Sir Robert Armstrong-Jones and Sir Frederick Mott.

### Fête at West Suffolk Hospital.

As the result of the Annual Hospital Fête held for the West Suffolk General Hospital, Bury St. Edmunds, on Bank Holiday, the sum of £1,250 has been handed to the Reconstruction Fund. This Fund was started in 1919, and has already provided new machinery in the laundry and kitchen, modern X-ray and electrical apparatus, and has also supplied the money for the erection of a nurses' hostel for thirty-one nurses. It is hoped that enough money may be forthcoming as the result of the Fête to supply some accommodation for private patients and a new out-patients' department, both of which are pressing needs. As the principal attraction of the Fête was a musical ride and display by the Life Guards—a necessarily large expense—it is very satisfactory to note so large a profit.

### More Accident Beds at King's College Hospital.

As the result of an offer of assistance by King Edward's Hospital Fund for London, fourteen additional beds for accident cases have been opened at King's College Hospital in order to prevent the hospital from being any longer obliged to send away, owing to lack of beds, ambulance cases which would otherwise have been admitted. The opening of these accident beds is the first result of the decision of the King's Fund to apply the legacies of the late Mr. and Mrs. John Wells, estimated at over £200,000, to assisting the hospitals of London with extensions, improvements and other special needs. The Fund anticipates, writes Mr. Bedwell, the House Governor of King's College, that the people of London will contribute the annual cost of maintaining these beds when the importance of its position in a part of the metropolis where accommodation is particularly needed is more fully realised.

